# Supplementary material for: The insidious degeneration of white matter and cognitive decline in Fabry disease
Source: PLoS One. 2025 Nov 17;20(11):e0325403. doi: 10.1371/journal.pone.0325403 (PMC12622807; doi:10.1371/journal.pone.0325403)
Supplement: S1 Fig — Age (a), sex (b), and ethnicity (c) are compared between participants with Fabry disease and controls. In (d), clinical characteristics are compared. htn = hypertension, chol = hypercholesterolemia, ns = not significant. (PDF) [file pone.0325403.s001.pdf]

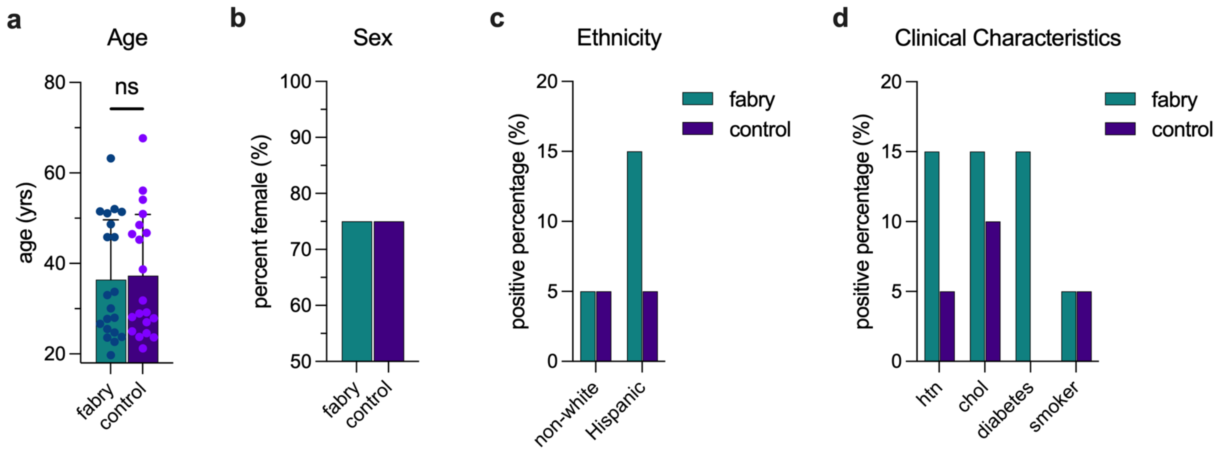

S1 Fig. Demographics and clinical characteristics of the case-control cohorts. Age (**a**), sex (**b**), and ethnicity (**c**) are compared between participants with Fabry disease and controls. In (**d**), clinical characteristics are compared. htn = hypertension, chol = hypercholesterolemia, ns = not significant
