# Supplementary material for: The insidious degeneration of white matter and cognitive decline in Fabry disease
Source: PLoS One. 2025 Nov 17;20(11):e0325403. doi: 10.1371/journal.pone.0325403 (PMC12622807; doi:10.1371/journal.pone.0325403)
Supplement: S2 Fig — In (a), a scatter plot of verbal IQ vs. age is shown along with regression lines. Although verbal IQ tended to decrease with age in both cohorts, the association was not significant (Pearson’s r). For Fabry (b) and controls (c), a range of verbal IQ values were present regardless of age and sex. Bars represent median values. (PDF) [file pone.0325403.s002.pdf]

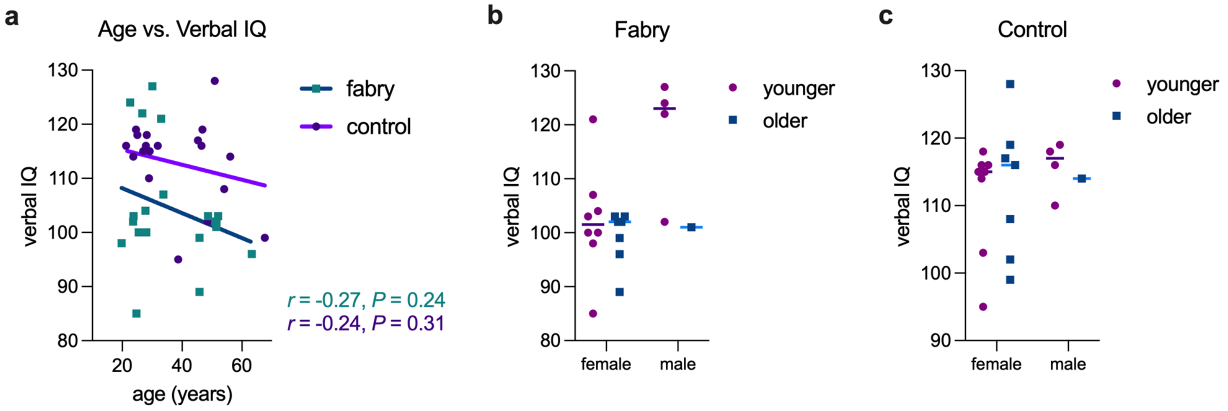

S2 Fig. Association between age and verbal intelligence quotient (IQ). In (**a**), a scatter plot of verbal IQ vs. age is shown along with regression lines. Although verbal IQ tended to decrease with age in both cohorts, the association was not significant (Pearson's *r*). For Fabry (**b**) and controls (**c**), a range of verbal IQ values were present regardless of age and sex. Bars represent median values.
