# Supplementary material for: The insidious degeneration of white matter and cognitive decline in Fabry disease
Source: PLoS One. 2025 Nov 17;20(11):e0325403. doi: 10.1371/journal.pone.0325403 (PMC12622807; doi:10.1371/journal.pone.0325403)
Supplement: S3 Fig — Years of education were similar between Fabry patients with and without an error during the Trail Making Test (Part A). ns = not significant. (PDF) [file pone.0325403.s003.pdf]

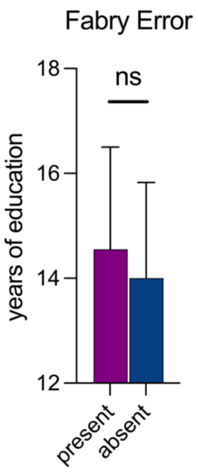

S3 Fig. Years of education in Fabry participants with an error during the Trail Making Test (Part A). Years of education were similar between Fabry patients with and without an error during the Trail Making Test (Part A). ns = not significant
