# Supplementary material for: The insidious degeneration of white matter and cognitive decline in Fabry disease
Source: PLoS One. 2025 Nov 17;20(11):e0325403. doi: 10.1371/journal.pone.0325403 (PMC12622807; doi:10.1371/journal.pone.0325403)
Supplement: S4 Fig — In (a), tables are shown for the blinded classification of case/control status by the study’s neuroradiologist using FLAIR, T2W, and MP-RAGE images. In (b), the same classification by the study’s neuroradiologist is used but the truth set was regrouped based on age (younger, < 40 yrs vs. older >40 yrs) rather than case/control status. In (a) and (b), P-values obtained using Fisher’s exact test are shown under each table. (PDF) [file pone.0325403.s004.pdf]

a

FLAIR		Case/Control: Control	Case/Control: Fabry
Radiology	Control	12	12
	Fabry	8	8

P > 0.99

T2W		Case/Control: Control	Case/Control: Fabry
Radiology	Control	8	10
	Fabry	12	10

P = 0.75

MP-RAGE		Case/Control: Control	Case/Control: Fabry
Radiology	Control	10	7
	Fabry	10	13

P = 0.52

b

FLAIR		Age: Younger	Age: Older
Radiology	Control	19	5
	Fabry	5	11

***P* = 0.004**

T2W		Age: Younger	Age: Older
Radiology	Control	13	5
	Fabry	11	11

P = 0.20

MP-RAGE		Age: Younger	Age: Older
Radiology	Control	14	3
	Fabry	10	13

***P* = 0.022**

S4 Fig. Contingency tables for case/control categorization by qualitative image interpretation. In (**a**), tables are shown for the blinded classification of case/control status by the study's neuroradiologist using FLAIR, T2W, and MP-RAGE images. In (**b**), the same classification by the study's neuroradiologist is used but the truth set was regrouped based on age (younger, <40 yrs vs. older >40 yrs) rather than case/control status. In (**a**) and (**b**), *P*-values obtained using Fisher's exact test are shown under each table.
