# Supplementary material for: The insidious degeneration of white matter and cognitive decline in Fabry disease
Source: PLoS One. 2025 Nov 17;20(11):e0325403. doi: 10.1371/journal.pone.0325403 (PMC12622807; doi:10.1371/journal.pone.0325403)
Supplement: S5 Fig — Representative reconstructed sagittal images from FLAIR (left) and MP-RAGE (right) images are shown off-center from the corpus callosum to provide a representation of brain tissues sampled. The dashed gray line outlines the field-of-view for each image. The shaded region superior to the solid gray line (left) and between the two solid gray lines (right) were isolated for histogram and volume analysis. Images are shown before the brain tissue mask was applied to show anatomic detail for reference. (PDF) [file pone.0325403.s005.pdf]

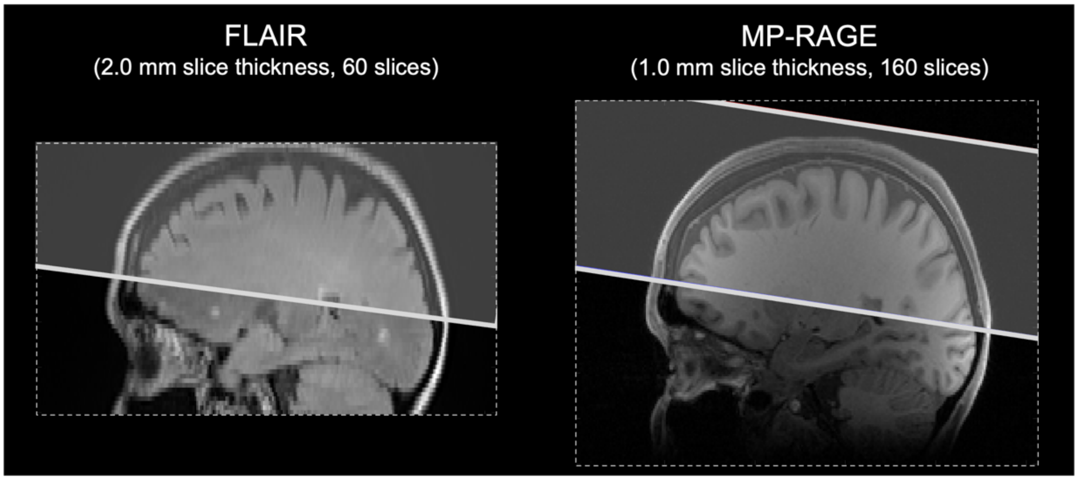

S5 Fig. Brain regions selected for histogram analysis. Representative reconstructed sagittal images from FLAIR (left) and MP-RAGE (right) images are shown off-center from the corpus callosum to provide a representation of brain tissues sampled. The dashed gray line outlines the field-of-view for each image. The shaded region superior to the solid gray line (left) and between the two solid gray lines (right) were isolated for histogram and volume analysis. Images are shown before the brain tissue mask was applied to show anatomic detail for reference.
