# Supplementary figures and images for: The insidious degeneration of white matter and cognitive decline in Fabry disease
Source: PLoS One. 2025 Nov 17;20(11):e0325403. doi: 10.1371/journal.pone.0325403 (PMC12622807; doi:10.1371/journal.pone.0325403)

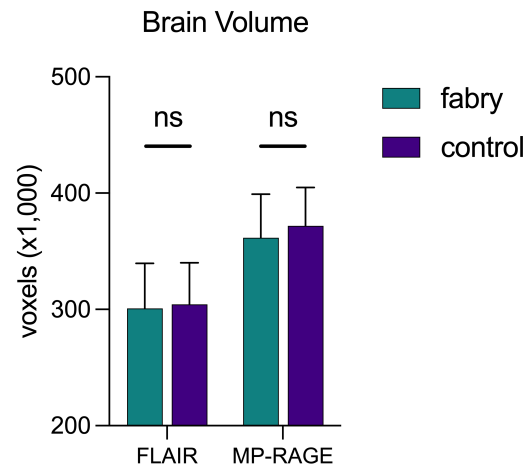

**S6 Fig. Comparison of structural MRI brain volumes.** ns = not significant

Supplement: S6 Fig — (PDF) [file pone.0325403.s006.pdf]
