# Supplementary material for: The insidious degeneration of white matter and cognitive decline in Fabry disease
Source: PLoS One. 2025 Nov 17;20(11):e0325403. doi: 10.1371/journal.pone.0325403 (PMC12622807; doi:10.1371/journal.pone.0325403)
Supplement: S7 Fig — Although Pearson’s correlation (r) was significant in the Fabry cohort, the controls followed a similar trend, and the difference between the slopes of the regression lines was not significant. (PDF) [file pone.0325403.s007.pdf]

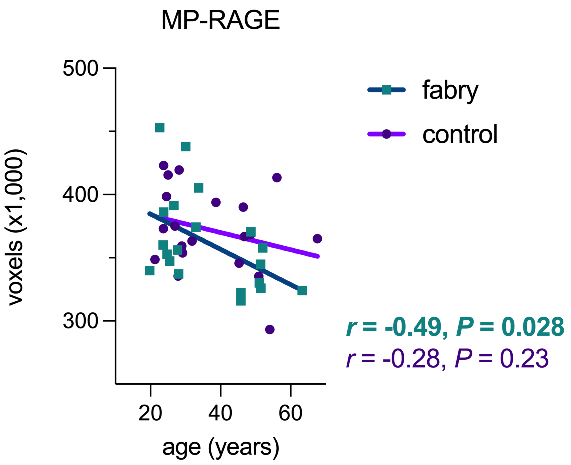

S7 Fig. Association between brain volume and age using MP-RAGE. Although Pearson's correlation (*r*) was significant in the Fabry cohort, the controls followed a similar trend, and the difference between the slopes of the regression lines was not significant.
