# Supplementary material for: The insidious degeneration of white matter and cognitive decline in Fabry disease
Source: PLoS One. 2025 Nov 17;20(11):e0325403. doi: 10.1371/journal.pone.0325403 (PMC12622807; doi:10.1371/journal.pone.0325403)
Supplement: S8 Fig — In (a), a comparison of MP-RAGE histograms using the normalized signal intensity (nSI) from the age-based first and fourth quartiles of healthy controls. The histograms for the MP-RAGE nSI from the younger adult (<40 yrs) Fabry and control cohorts are shown in (b). (PDF) [file pone.0325403.s008.pdf]

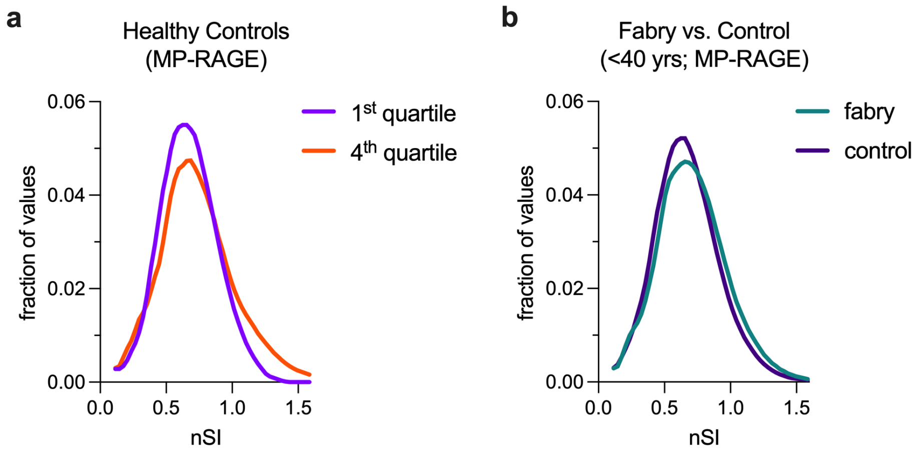

S8 Fig. Comparison of histograms using MP-RAGE. In (**a**), a comparison of MP-RAGE histograms using the normalized signal intensity (nSI) from the age-based first and fourth quartiles of healthy controls. The histograms for the MP-RAGE nSI from the younger adult (<40 yrs) Fabry and control cohorts are shown in (**b**).
