# Supplementary material for: The insidious degeneration of white matter and cognitive decline in Fabry disease
Source: PLoS One. 2025 Nov 17;20(11):e0325403. doi: 10.1371/journal.pone.0325403 (PMC12622807; doi:10.1371/journal.pone.0325403)
Supplement: S9 Fig — In (a), the comparison of volumes between Fabry and controls is shown. In (b), the association between brain volumes and age is shown. Although Pearson’s correlation (r) was significant in controls, the Fabry cohort followed a similar trend. ns = not significant. (PDF) [file pone.0325403.s009.pdf]

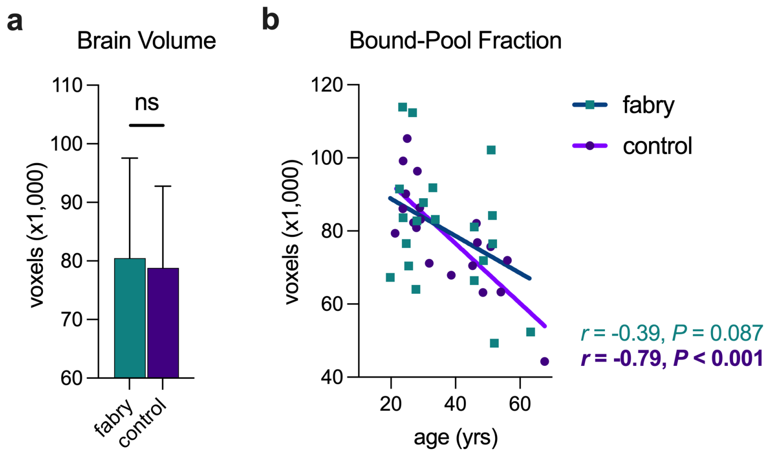

S9 Fig. Bound-pool fraction volumes and association with age. In (**a**), the comparison of volumes between Fabry and controls is shown. In (**b**), the association between brain volumes and age is shown. Although Pearson's correlation (*r*) was significant in controls, the Fabry cohort followed a similar trend. ns = not significant
