# Supplementary material for: The insidious degeneration of white matter and cognitive decline in Fabry disease
Source: PLoS One. 2025 Nov 17;20(11):e0325403. doi: 10.1371/journal.pone.0325403 (PMC12622807; doi:10.1371/journal.pone.0325403)
Supplement: S10 Fig — Fractional anisotropy (a) and mean diffusivity (b) are compared based on presence/absence of WMLs. Associations between Fazekas score and fractional anisotropy (FA; c) and mean diffusivity (MD, d) show significant correlations in Fabry and strong trends in controls. (PDF) [file pone.0325403.s010.pdf]

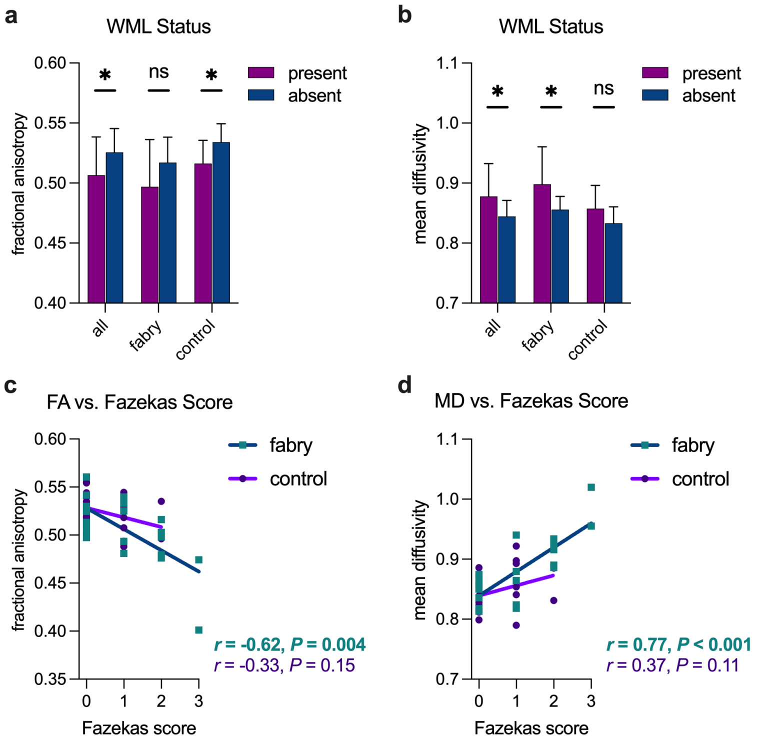

S10 Fig. White matter lesions (WMLs) and effects on diffusion-based parameters. Fractional anisotropy (**a**) and mean diffusivity (**b**) are compared based on presence/absence of WMLs. Associations between Fazekas score and fractional anisotropy (FA; **c**) and mean diffusivity (MD, **d**) show significant correlations in Fabry and strong trends in controls.
