# Supplementary material for: The insidious degeneration of white matter and cognitive decline in Fabry disease
Source: PLoS One. 2025 Nov 17;20(11):e0325403. doi: 10.1371/journal.pone.0325403 (PMC12622807; doi:10.1371/journal.pone.0325403)
Supplement: S11 Fig — In (a), verbal IQ is compared between the presence/absence of a white matter lesion (WML). The association between verbal IQ and Fazekas score is shown in (b). Although a significant association (Pearson’s r) between verbal IQ and corpus callosum body (CCB) volume was present in Fabry (c), the significance resolved once the volume was normalized (nCCB) for age (d). Significant associations were not observed between age and fractional anisotropy (e) and mean diffusivity (f). (PDF) [file pone.0325403.s011.pdf]

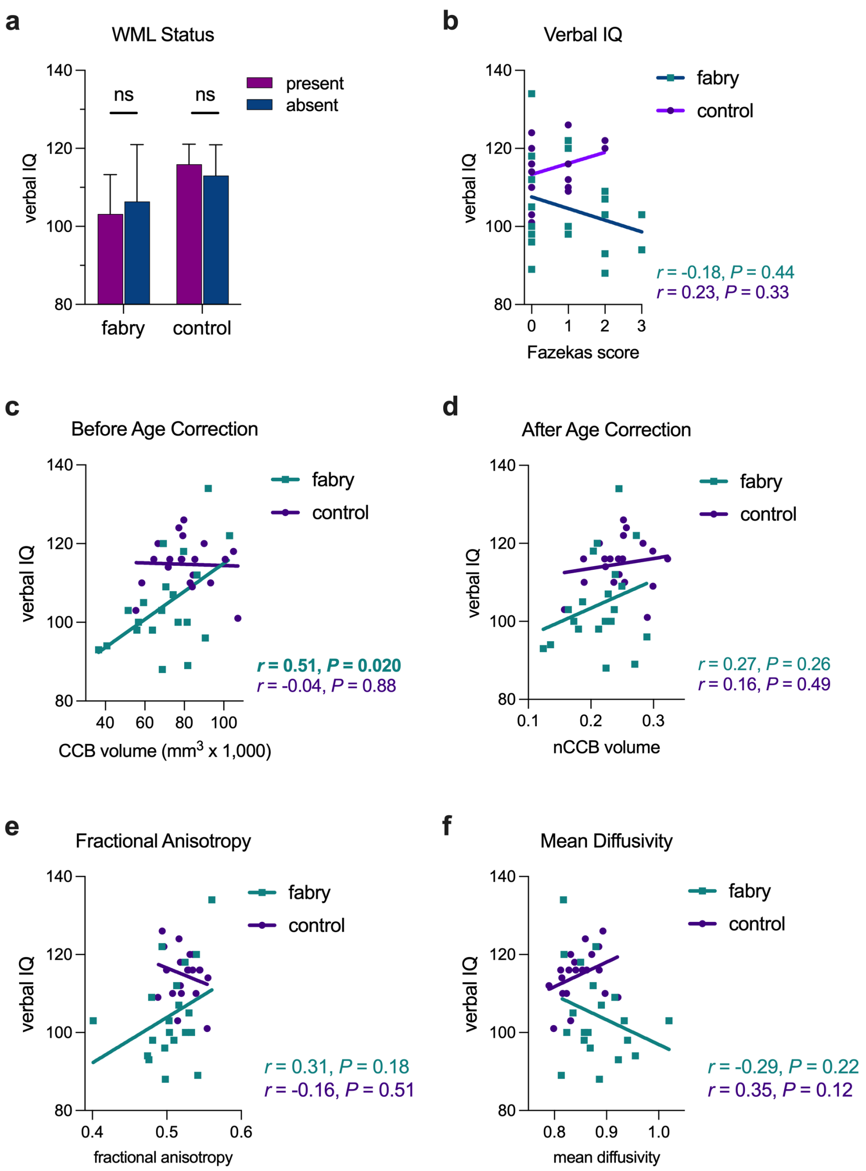

S11 Fig. Comparisons between verbal IQ and imaging metrics. In (**a**), verbal IQ is compared between the presence/absence of a white matter lesion (WML). The association between verbal IQ and Fazekas score is shown in (**b**). Although a significant association (Pearson's *r*) between verbal IQ and corpus callosum body (CCB) volume was present in Fabry (**c**), the significance resolved once the volume was normalized (nCCB) for age (**d**). Significant associations were not observed between age and fractional anisotropy (**e**) and mean diffusivity (**f**).
