# Supplementary material for: The insidious degeneration of white matter and cognitive decline in Fabry disease
Source: PLoS One. 2025 Nov 17;20(11):e0325403. doi: 10.1371/journal.pone.0325403 (PMC12622807; doi:10.1371/journal.pone.0325403)
Supplement: S12 Fig — Fazekas score (a), normalized corpus callosum body (nCCB) volume (b), fractional anisotropy (c), and mean diffusivity (d) are compared in the Fabry cohort based on the presence/absence of error during the Trail Making Test (Part A). ns = not significant. (PDF) [file pone.0325403.s012.pdf]

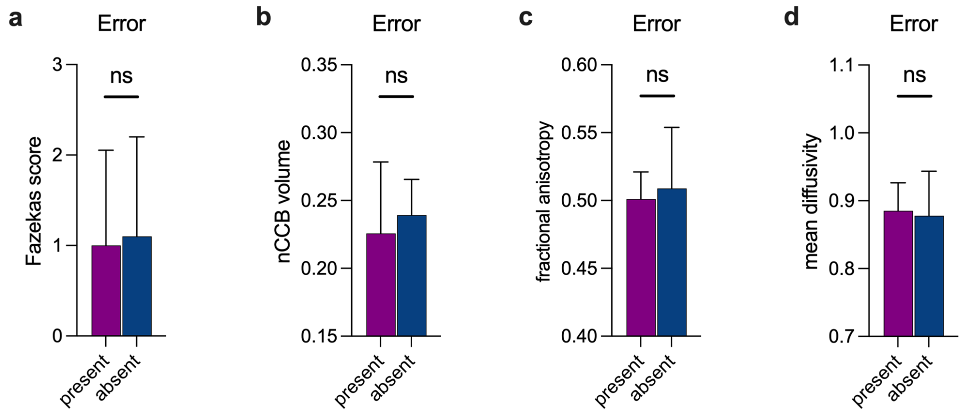

S12 Fig. Comparisons between imaging metrics and executive function in the Fabry cohort. Fazekas score (**a**), normalized corpus callosum body (nCCB) volume (**b**), fractional anisotropy (**c**), and mean diffusivity (**d**) are compared in the Fabry cohort based on the presence/absence of error during the Trail Making Test (Part A). ns = not significant.
